# Redirection of Urgent Geriatric Care: Diagnostics and Treatment Parallel to the Emergency Department

**DOI:** 10.3390/jcm15082989

**Published:** 2026-04-15

**Authors:** Lennaert A. R. Zwart, Nikki M. F. Noorda, Chantal H. N. van Dijk, Naomi Hoekstra-Zuidema, Margreet G. Kamp-Glas, Anna C. M. Mulder, Judella O. Daal

**Affiliations:** Department of Geriatric Medicine, Dijklander Hospital, 1624 NP Hoorn, The Netherlands; n.m.f.noorda@dijklander.nl (N.M.F.N.); c.h.n.vandijk@dijklander.nl (C.H.N.v.D.); n.zuidema@dijklander.nl (N.H.-Z.); m.g.kamp-glas@dijklander.nl (M.G.K.-G.); a.c.m.mulder@dijklander.nl (A.C.M.M.); j.o.daal@dijklander.nl (J.O.D.)

**Keywords:** frailty, multimorbidity, emergency department, redirection of care, organisation of care

## Abstract

**Objectives**: Complex patients in need of an urgent medical assessment can contribute to crowding in the Emergency Department (ED). Optimising access to geriatric expertise for this patient group is known as ‘Geriatric Emergency Departments’. **Methods**: A parallel care pathway was designed to redirect frail older patients to an Urgent Geriatric Care (UGC) service rather than the ED. The UGC has access to the diagnostic facilities of the ED. This descriptive analysis reports on delivered care, diagnostics, admissions rates, discharge policy, and 30-day and 6-month outcomes concerning hospital (re)admissions, ED visits, and mortality. **Results**: 269 patients were analysed. The median age was 83 years, 68% had polypharmacy, 51% cognitive disorders, and 83% a gait disturbance. A median of four conclusions was drawn per patient. Evaluation at the UGC often leads to medication regimen changes (81%), initiation or expansion of care at home (46%), and initiation of dementia care (18%). The hospital admission rate was 13%; the rate of ED visits within 30 days was 5% and, within 6 months, an additional 16%; the rate of hospital readmissions within 30 days was 7%, and 11% after 6 months. The mortality rates were 9% within 30 days and 12% within 6 months. **Conclusions**: Evaluation of patients at the UGC led to a high degree of medication regimen changes, initiation of care at home, and multiple conclusions or diagnoses per patient. Readmission or revisiting rates were low. A direct comparison to care delivered at the ED should be made in a future study.

## 1. Introduction

Expected demographic changes will lead to an increasingly multi-morbid and complex population of older patients visiting the Emergency Department (ED). Frailty contributes to the complexity of this patient population and is associated with worse patient outcomes [1,2]. Furthermore, the presenting symptoms can be non-descriptive, such as a fall, in which case a full geriatric work-up would be preferable but is often not feasible or instantly available within the ED setting. Addressing and recognising geriatric giants, such as cognitive impairment, delirium, impaired mobility, and dependence on others, can cause delays in treatment and prolonged stays at the ED [2,3]. These issues contribute to ED crowding. An extensive analysis of ED visits among patients aged 70 years and older in Melbourne found that close to 14% of ED visits were potentially avoidable [4]. Most of these visits (88%) were initiated by patients themselves, and close to 60% did not need further hospital treatment [4]. Wounds and injuries after a fall were the most common reason for the ED visit, and during the 4-year study period, the number of all ED visits for this age group increased by more than 12% [4].

The upcoming challenges within urgent care for frail older people are well recognised, and a variety of initiatives have been developed to address them [2,5,6,7]. At the core of these solutions is a multidisciplinary team with geriatric expertise, who can either blend into the ED or work in a dedicated area for older patients within the EDs [5,6,7].

In this descriptive analysis, we will describe an Urgent Geriatric Care service (UGC service) implemented in 2023 at our non-academic teaching hospital. The delivered care and outcomes will be described, along with relevant outcomes such as recurrent ED visits, hospital admissions, and mortality.

## 2. Methods

### 2.1. The Urgent Geriatric Care Service

The UGC service attempts to address the need for an urgent assessment of frail older people by performing a Comprehensive Geriatric Assessment (CGA) with a specialised geriatric nurse and geriatrician, with support from the care liaison if necessary. Patients are evaluated in the adjacent rooms next to the ED, which functions as a General Practitioner’s out-of-office-hours service during the evening and overnight, but was not in use during office hours. This has the logistical advantage of providing easy access to the ED’s diagnostic facilities and does not require major renovation since the rooms were already equipped for patient care. The UGC service is situated in a non-academic teaching hospital, and currently, two patients can be evaluated per day. The UGC service is financially organised and billed as a regular outpatient clinic; the geriatrician on duty at the UGC service combines these tasks with in-hospital consultations. In the Netherlands, when a patient visits the ED, the geriatrician on duty can be consulted for advice on the ED in a supervisory role. In contrast to the UGC service, the geriatrician is often not available to personally assess a patient in the ED.

**Figure 1:**
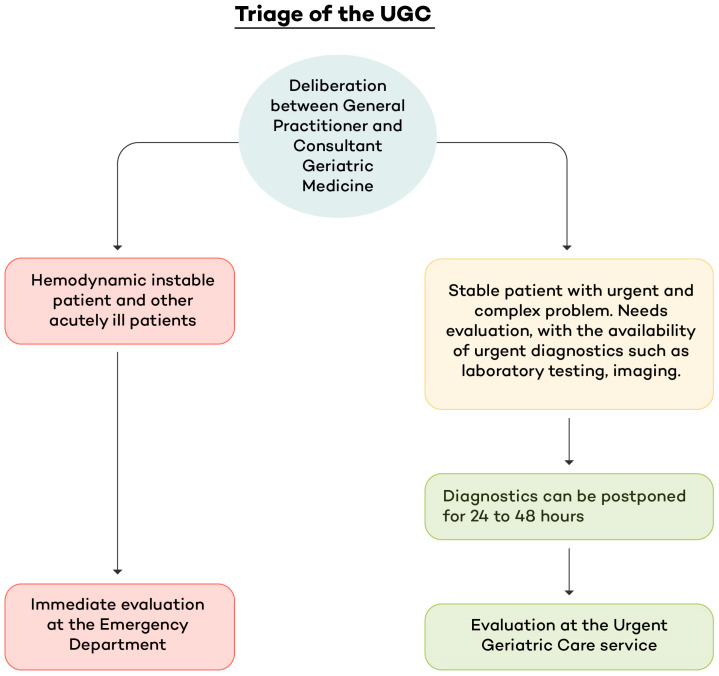
Triage of patients in the emergency department or urgent geriatric care service. Abbreviations: UGC, Urgent Geriatric Care service.

Patient selection and triage are visualised in Figure 1. Triage and planning are performed by the geriatrician on duty, in consultation with the general practitioner (GP) who calls about a patient. There is no age prerequisite; but generally, referred patients are above 70 years old. Suitability is mostly driven by ruling out reasons for immediate assessment, such as a high likelihood of the need for hospital admission, suspected sepsis, hemodynamically unstable patients, or patients in need of primary assessment by other specialities than geriatrics, for example, a suspicion of an acute stroke, or acute coronary syndrome. If a patient is hemodynamically stable but has the need for an urgent evaluation of a complex problem, preferably with access to directly available laboratory diagnostics and/or imaging, and can wait to be evaluated within 24 to 48 h, then the patient is suitable and is planned for evaluation at the UGC service. After triage, the geriatrician on duty anticipates and prepares the necessary diagnostics, already ordering laboratory analyses, imaging, and provides instructions for further evaluation, such as an electrocardiogram (ECG) and cognitive screening.

Within the UGC service, the CGA is performed by the specialised geriatric nurse and geriatrician. Depending on the presenting issue and findings, other medical specialists can be consulted and made available to perform a consultation at the UGC service. Laboratory testing and imaging are performed, analysed, and reported with priority, in a manner similar to ED diagnostics. The combination of the CGA, diagnostics and its analysis and reporting, is performed between 8:30 and 12:00 in the morning. Then the care liaison can join to determine whether care at home needs to be requested or expanded, and a conference call with the referring GP is made to inform them of the findings and invite them to consider the proposed treatment and guidance plan. Finally, the patients are informed of the findings and the treatment proposal, during which the nurse provides a summary in writing of the visit in easily understandable terms. Patients are then discharged home or can be admitted to the hospital or other facilities for treatment.

### 2.2. Frailty Assessment

For all patients, the Charlson Comorbidity Index (CCI) will be calculated [8]. Also, the Nurse-Directed Frailty Assessment (NDFA) tool will be applied [9]. The NDFA was recently validated against a Frailty Index (FI) based on the Accumulation of Deficits model and showed performance similar to the FI in identifying patients at risk for ED visits, unplanned hospital admissions, and mortality at 6-month and 1-year follow-up [9,10,11]. The presence of polypharmacy was defined as the use of 5 or more prescription drugs [12].

### 2.3. Primary Outcomes

This analysis primarily serves as a description of the UGC service, including the performed diagnostics, discharge conclusions and destinations, treatment decisions, rates of revisiting the ED, hospital readmissions, and mortality. The number of visits to the UGC service and occupancy rate will be reported. Frequencies of utilisation of diagnostics will be reported, including laboratory testing, conventional X-rays and imaging, performance of ECG, cognitive screening, and consultations with other medical specialities. The suitability of the case for evaluation on the UGC service will be assessed. Cases that follow the triage flowchart are deemed suitable. If patients are evaluated at the UGC service after more than 2 working days, these cases are deemed unsuitable. All remaining cases are evaluated independently by two geriatricians (CD and NN). Outcomes of the CGA, such as the conclusions, final diagnoses, and treatment decisions, will be described, as will be the rate of admission to the hospital or other facilities. Recurrent visits to the ED, hospital admissions, and mortality within 30 days and 6 months will be reported.

### 2.4. Data Extraction and Statistical Analysis

The study complies with the Declaration of Helsinki, and the protocol was approved by the Institutional Review Board of the Dijklander Hospital. As data was collected retrospectively and had no influence on treatment decisions or patient selection, informed consent for the use of medical data was deemed not necessary. A hospital-wide opt-out for the use of medical data is available to patients; if exercised, their data was not included in this analysis. Frequencies, occupancy rates, and mortality will be reported as a percentage. The number of recurrent ED visits and hospital admissions is expected to have a skewed distribution and will be reported as a median count value, with an interquartile range. Data were analysed in SPSS for Windows, version 20. The manuscript was written without the use of Large Language Models.

## 3. Results

### 3.1. Assessment at the UGC Service

In total, 279 patients visited the UGC service 282 times, 3 patients visited twice; only the first visit is included in this analysis, which comprises the first full calendar year that the UGC service was in operation, and excluding the starting months of November and December 2023. Ten patients opted out of the use of their medical data and were not included in the analysis; a total of 269 patient visits to the UGC service were analysed. There was one patient who passed away while on route to the UGC service, whose suspected cause of death was a myocardial infarction. The baseline demographic data are shown in Table 1. In summary, the median age was 83 years (IQR 10, ranging from 63 to 104, and 94.8% were 70 years or older). The data was normally distributed; 156 (58.0%) were female, and polypharmacy was present in 183 (68%) patients. Cognitive disorders and dependence on others for instrumental daily activities were very prevalent, 138 (50.1%) and 225 (83.5%), respectively. Both the CCI and the NDFA were normally distributed, with median CCI and NDFA scores of 7 (IQR 3) points and 5 (IQR 2) points, respectively.

There were 400 timeslots available on the UGC service; the occupancy rate was 70.5%. In 27 (10.0%) cases, a timeslot was used for a patient who did not follow the intended triage flow. In 12 (4.5%) cases, the assessment was conducted more than 3 working days after the event. In 15 (5.6%) cases, an available timeslot was utilised to assess a patient with a regular outpatient clinic referral. After review, 10 (3.7%) of these cases were deemed suitable for the UGC service, 3 (1.1%) were deemed not suitable, and there was disagreement in 2 cases (0.7%). The diagnostics that were performed are shown in Table 2. In summary, more than 95% of the patients underwent laboratory testing and an electrocardiogram (ECG). Radiology investigations were frequently made, with the majority of patients undergoing a chest X-ray (49.8%), computer tomography (CT) scan of the brain (41.3%), or CT of the abdomen (20.8%). Supplementary Table S1 gives an overview of other types of radiology investigations that were made. Cognitive screening was performed in 123 (45.7%) patients, and deliberation or assessment by another consultant at the UGC service took place in 73 (27.1%) patients.

### 3.2. Outcomes of the UGC

In most patients, multiple conclusions and diagnoses could be made, with a median of 4 (IQR 1) issues per patient. The five most frequent medical diagnoses were medication related complaints (80 patients, 29.7%), infections (65 patients, 24.2%), new diagnosis of dementia or mild cognitive impairment (65 patients, 24.2%, and 45 patients, 16.7%, respectively), anaemia (43 patients, 16.0%), and cerebral vascular damage (35 patients, 13.0%). Furthermore, a delirium was seen in 44 patients (16.1%), a gait disorder in 187 (69.5%), orthostatic hypotension in 117 (43.5%), malignancies in 26 (9.7%), and atrial fibrillation in 6 patients (2.2%). Discharge diagnoses are summarised in Supplementary Table S2.

Changes in medication were frequently made; in 217 patients (80.7%), medication was altered, and in 101 patients (37.5%), more than one drug was changed. New drugs were initiated in 180 patients (66.9%), dosage or regimen changes were applied in 66 patients (24.5%), and medication was stopped in 90 patients (33.5%). There were 36 patients (13.4%) admitted to the hospital from the UGC service, 5 (1.9%) were redirected at arrival from the UGC service to the Emergency Department, and 15 (5.6%) were admitted in the ‘Ouderenkliniek’ which is a local initiative of cooperation of Geriatricians and Elderly Care physicians to deliver hospital-like treatments such as intravenous antibiotics or diuretics on the Geriatric Rehabilitation ward, instead of a hospital admission. There were 8 (3.0%) patients admitted to a Rehabilitation facility, and 15 (5.6%) were admitted to a nursing home. For 124 patients (46.1%), extra care at home, such as physiotherapy, dietary interventions, and/or nurses to aid with personal hygiene, was either organised or applied for, depending on the availability of staff of local home care organisations. Treatment decisions and discharge policy are shown in Table 3.

In Table 4, the outcomes at 30 days and 6 months are summarised. Within 30 days, 25 patients (9.3%) died; the most common cause of death was a malignancy. Between 30 days and 6 months, an additional 33 patients (12.3%) died, of which the most frequent known cause of death was malignancies. Supplementary Table S2 shows all causes of death within 30 days and 6 months.

Within the first 30 days after the UGC service visit, 21 patients (4.8%) visited the ED once, of whom 4 patients (1.5%) visited the ED multiple times. There were 18 patients (6.7%) with an unplanned hospital admission (other than the 36 admissions that took place directly from the UGC service), of whom 4 patients (1.5%) had multiple unplanned hospital admissions within 30 days. Follow-up data between 30 days and 6 months for a total of 244 patients was analysed. Of these patients, 42 (15.6%) visited the ED, of whom 12 (4.4%) visited the ED multiple times. There were 29 patients (10.8%) with unplanned hospital admission, of whom 4 patients (1.6%) had multiple unplanned hospital admissions.

Within 30 days, a fall was the most frequent reason for an ED visit (4 patients, 15.4%), followed by heart failure (3 patients, 11.5%). Between 30 days and 6 months, a fall was the most frequent reason for an ED visit (11 patients, 26.2%); 3 patients had a hip fracture (7.1%), and 3 patients had other fractures (7.1%). Supplementary Table S3 shows all reasons for ED visits.

## 4. Discussion

This article describes the Urgent Geriatric Care service, which aims to deliver geriatric expertise to older patients by redirecting older patients in need of urgent assessment away from the ED, to a parallel program. It could relieve crowding of the ED by the increasing number of older and complex patients who visit the ED. The patients who visited the UGC service are indeed a cohort with more threatening and urgent issues than the general geriatric outpatient clinic population. Compared with recent studies in other Dutch clinics, both single-centre and multi-centre, including fall clinics and memory clinics, the UGC cohort is older, with a median of 83 years, whereas the outpatient cohorts range from 78 to 81 years [9,13,14,15]. Also, patients at the UGC service have a higher NDFA score, with a median of 5 points compared with 3 points in the original validation study, suggesting that patients at the UGC service are frail to a higher degree than those at the general outpatient clinic [9]. Furthermore, polypharmacy was highly prevalent in patients at the UGC service, in 68%, and ranged in the outpatient cohorts from 52 to 70% [9,13,14,15]. A higher incidence of atrial fibrillation was seen, as compared to outpatient cohorts [14,15]. But foremost, the mortality rate illustrates clearly the difference between the UGC and a general geriatric outpatient cohort, suggestive of the UGC cohort suffering from more threatening issues. For the UGC cohort, the 30-day and 6-month mortality rates were 9.3 and 12.3%, whereas the NDFA study at the same hospital (inclusion period January 2021 to October 2022) reported a 1.6% 6 months, and 4.8% 1 year mortality rate in a outpatient cohort [9].

At the moment, a comparison of our hospital’s ED admission, readmission, and re-visiting rates is not available, but is in preparation. For perspective, the UGC cohort could be compared to the literature on other ED cohorts. Comparisons have to be made with caution, however, as a selection of patients who do not need immediate assessment or treatment is made beforehand. Before visiting the UGC service, the general practitioner and geriatrician assess the urgency of the situation and refer acutely ill patients directly to the ED. Naturally, a high proportion of these patients are admitted to the hospital. Therefore, readmission rates serve as a more appropriate outcome to evaluate the delivered care at the UGC service. The reported readmission and revisiting rates in this analysis seem similar to those reported in an article on the InteRAI study performed in Switzerland in 2020 [16]. The patient characteristics of the cohort in the InteRAI study are very much alike those visiting the UGC service, with a similar age, proportion with cognitive impairment, gait disturbances, and polypharmacy. In this study, the authors reported an admission rate after an ED visit of 55%, which was much higher than the 13.4% observed in the UGC cohort. As stated, a difference of this magnitude was to be expected, as patients in need of acute care and treatment would be presented directly to the ED rather than the UGC service, decreasing the likelihood of hospital admission for the remaining patients after the UGC service visit. For revisiting the ED within 30 days, a similar pattern can be observed: a combined ED and hospital readmission rate of 11.4% in the InteRAI study, and ED visit rates of 7.8% and hospital admission rates of 6.7% after visiting the UGC service [16]. Although the current analysis does not allow a direct comparison of care delivered in the UGC service versus the ED, the observations do not show higher rates of mortality or readmissions after discharge from the UGC service.

The UGC approach’s strengths lie in its multidisciplinary team with geriatric expertise at its core. The service implements the Geriatric Emergency Department (GED) principles [5,6,7]. The UGC service is tailored to local circumstances, taking into account available facilities, staff, and organisational structures. It is located in the treatment rooms adjacent to the ED, which are not used during the day, optimising access to diagnostic facilities of the ED and making renovations or expansion of the existing ED unnecessary. Tailoring the pathway in this manner to the local opportunities greatly supported its swift implementation. A formal ‘return on investment’ analysis was not performed. The UGC service is financially organised and billed as a regular outpatient service. It allocates a geriatrician and a geriatric nurse to the UGC service, who also perform in-hospital consultations on different wards in the remaining time of the day. The occupancy rate of 70.5% reflects the on-demand nature of the UGC. After the first evaluation of the UGC, from 2025 onward, if unutilized spots remain, patients from the waiting list after a regular referral are invited and evaluated instead to make best use of the capacity. The aim was to redirect patients with complex but not life-threatening problems away from the ED and address their needs with a full CGA by the geriatric care team, thereby providing more patient-centred care and reducing crowding of the ED [2]. A limitation is that a direct comparison with care delivered in the ED cannot be made at the moment. To determine the impact of the UGC service on recurrent ED visits and unplanned hospitalisations, or on utilised diagnostics, a comparison with a cohort of patients who were evaluated in the ED is warranted. The approach might not be generalisable to other hospitals or healthcare systems; however, it is likely that in many settings, frail older people are assessed in the ED without requiring immediate medical treatment, and an alternate approach tailored to local circumstances could be of added value.

## 5. Conclusions

We can conclude that indeed a cohort of patients in need of more urgent care than at the general outpatient clinic was referred and selected for assessment at the UGC service. In the current analysis, a low rate of ED or hospital readmission was seen. A direct comparison with the findings and outcomes of an ED cohort is necessary, and in preparation. Potentially, the UGC service could reduce crowding in the ED while simultaneously providing a higher level of geriatric expertise to the older patient in need of urgent evaluation. With the growing demand for Emergency Department care, it is necessary to rethink which patients should be evaluated there and whether such evaluations should be immediate. These findings provide encouragement to redesign patient flow, as a full CGA delivers a nuanced assessment and treatment plan without high readmission or revisiting rates.

## Figures and Tables

**Table 1:** Demographics.

	Total *n* = 269 Patients
Female, *n* (%)	156 (58.0)
Age in years, median (IQR)	83 (10)
Charlson Comorbidity Index, median (IQR)	7 (3)
Nurse-Directed Frailty Assessment, median (IQR)	5 (2)
Polypharmacy, *n* (%)	183 (68.0)
Cognitive disorders	
MCI, *n* (%)	50 (18.6)
Dementia, *n* (%)	88 (32.7)
Gait disorder, *n* (%)	223 (82.9)
Dependence in ADL, *n* (%)	156 (58.0)
Dependence in iADL, *n* (%)	225 (83.6)

Abbreviations: IQR, interquartile range; MCI, mild cognitive impairment; ADL, activities of daily living; iADL, instrumental activities of daily living.

**Table 2:** Performed diagnostics.

	Total *n* = 269 Patients
Laboratory diagnostics (blood and/or urine), *n* (%)	266 (98.9)
ECG, *n* (%)	262 (97.4)
Radiology	
Chest X-ray, *n* (%)	134 (49.8)
Neck or spine X-ray, *n* (%)	19 (7.1)
CT cerebrum, *n* (%)	111 (41.3)
CT thorax, *n* (%)	43 (16.0)
CT abdomen, *n* (%)	56 (20.8)
Other, *n* (%)	40 (14.9)
Cognitive screening, *n* (%)	123 (45.7)
Deliberation with other consultants, *n* (%)	73 (27.1)

Abbreviations: ECG, electrocardiogram; CT, computer tomography.

**Table 3:** Treatment decisions and discharge policy.

	Total *n* = 269 Patients
Change in medication, *n* (%)	217 (80.7)
Initiation of new medication, (*n* %)	180 (66.9)
Dosage or regimen change in medication already in use, *n* (%)	66 (24.5)
Deprescribing, *n* (%)	90 (33.5)
Multiple medication alterations, *n* (%)	101 (37.5)
Further cognitive investigations, *n* (%)	12 (4.5)
Case manager, *n* (%)	47 (17.5)
Further radiology diagnostics, *n* (%)	39 (14.5)
Follow-up on outpatient Geriatric Medicine clinic, *n* (%)	95 (35.3)
Referral to other outpatient clinic, *n* (%)	59 (21.9)
Hospital admission, *n* (%)	36 (13.4)
Admission to the Ouderenkliniek, *n* (%)	15 (5.6)
Geriatric Rehabilitation programme, *n* (%)	8 (3.0)
Nursing home admission, *n* (%)	15 (5.6)
Hospice care, *n* (%)	5 (1.9)
Care at home, *n* (%)	124 (46.1)

**Table 4:** Outcomes at 30 days and 6 months.

Thirty-Day Follow-Up		Six-Month Follow-Up	
	Total *n* = 269 patients		Total *n* = 244 patients
Mortality, *n* (%)	25 (9.3)	Mortality, *n* (%)	33 (12.3)
ED visits, *n* (%)	21 (4.8)	ED visits, *n* (%)	42 (15.6)
Multiple ED visits, *n* (%)	4 (1.5)	Multiple ED visits, *n* (%)	12 (4.4)
Hospital admissions, *n* (%)	18 (6.7)	Hospital admissions, *n* (%)	29 (10.8)
Multiple hospital admissions, *n* (%)	4 (1.5)	Multiple hospital admissions, *n* (%)	4 (1.5)

Abbreviations: ED, Emergency Department.

## Data Availability

The dataset used and/or analysed during the current study is available from the corresponding author on reasonable request.

## Supplementary Materials

The following supporting information can be downloaded at: https://www.mdpi.com/article/10.3390/jcm15082989/s1, Table S1: Overview of radiology investigations; Table S2: Diagnoses and conclusions; Table S3: Causes of death.
