# Supplementary material for: Redirection of Urgent Geriatric Care: Diagnostics and Treatment Parallel to the Emergency Department
Source: J Clin Med. 2026 Apr 15;15(8):2989. doi: 10.3390/jcm15082989 (PMC13116490; doi:10.3390/jcm15082989)
Supplement: Supplementary file 1 [file jcm-15-02989-s001.zip › jcm-4181368-supplementary.pdf]

Supplementary Tables S1–S3

Supplementary Table S1. Overview of radiology investigations

	Total n = 269 patients
Abdominal ultrasound	12 (4.5)
CT neck, n (%)	5 (1.9)
Pelvic X ray	5 (1.9)
Mammography	3 (1.2)
MRI cerebrum	3 (1.2)
Facial ultrasound (arteritis temporalis)	2 (0.8)
Foot X ray	2 (0.8)
CT Intravenous Pyelography	1 (0.4)
CT upper leg	1 (0.4)
CT lumbar spine	1 (0.4)
CT pelvis	1 (0.4)
CT aorta	1 (0.4)
Hand X ray	1 (0.4)
MRI lumbar spine	1 (0.4)
Shoulder X ray	1 (0.4)

Abbreviations: CT, computer tomography; MRI, magnet resonance imaging.

Supplementary Table S2. Diagnoses and conclusions

	Total n = 269 patients
Gait disorder, (n %)	187 (69.5)
Accumulation of morbidities, (n %)	164 (61.0)
Orthostatic hypotension, (n %)	117 (43.5)
Medication related, (n %)	80 (29.7)
Infection, (n %)	65 (24.2)
Dementia, (n %)	65 (24.2)
Substance abuse, n (%)	52 (19.3)
Mild Cognitive Impairment, (n %)	45 (16.7)
Delirium, (n %)	44 (16.4)
Anemia, (n %)	43 (16.0)
Cerebral vascular damage, (n %)	35 (13.0)
Heart Failure, (n %)	26 (9.7)
Fracture or osteoporosis, (n %)	26 (9.7)
Malignancy, (n %)	26 (9.7)
Polyneuropathy, (n %)	25 (9.3)
Constipation, (n %)	19 (7.1)
Malnourishment, (n %)	18 (6.7)
Depression, (n %)	16 (5.9)
Stroke, (n %)	13 (4.8)
Suspected malignancy	10 (3.7)
Psychosis, (n %)	8 (3.0)
Atrial fibrillation	6 (2.2)
Anxiety disorder, n (%)	6 (2.2)
Angina pectoris, (n %)	4 (1.5)
Auto immun or neoplastic phenomona, (n %)	4 (1.5)
Catatonia, (n %)	2 (0.7)

Supplementary Table S3. Causes of death

	Within 30 days, n = 25	Between 30 days and 6 months, n = 33
Malignancy	9	10
Infection	2	1
Peripheral arterial disease	1	0
Heart failure	1	1
Myocardial infarct	1	0
Intracranial haemorrhage	1	1
Pulmonary embolism	1	0
Gastro intestinal bleeding	1	0
Dementia related	1	1
Unknown	7	19
Euthanasia	0	1
